# Impact of concomitant obstructive sleep apnea on pulmonary involvement and main pulmonary artery diameter in adults with scleroderma

**DOI:** 10.1007/s11325-020-02059-4

**Published:** 2020-04-13

**Authors:** Tugce Yakut, Baran Balcan, Sait Karakurt, Haner Direskeneli, Yasemin Yalcinkaya, Yüksel Peker

**Affiliations:** 1 Department of Allergology and Immunology, Süreyyapasa Chest Diseases and Chest Surgery Training & Research Hospital, Istanbul, Turkey; 2 Department of Pulmonary Medicine, Marmara University, School Medicine, Istanbul, Turkey; 3 Department of Rheumatology, School of Medicine, Marmara University, Istanbul, Turkey; 4 Department of Pulmonary Medicine, School of Medicine, Koc University, Koc University Hospital, Davutpasa cad, No. 4, Zeytinburnu, TR-34010 Istanbul, Turkey; 5 Department of Clinical Sciences, Respiratory Medicine and Allergology, Faculty of Medicine, Lund University, Lund, Sweden; 6 Sahlgrenska Academy, University of Gothenburg, Sweden, Gothenburg, Sweden; 7 Division of Pulmonary, Allergy, and Critical Care Medicine, University of Pittsburgh School of Medicine, Pittsburgh, PA USA

**Keywords:** Obstructive sleep apnea, Scleroderma, Pulmonary involvement, Pulmonary hypertension

## Abstract

**Purpose:**

Pulmonary involvement is common in adults with scleroderma. The effect of concomitant obstructive sleep apnea (OSA) on risk for pulmonary hypertension in scleroderma is unknown. An enlarged main pulmonary artery diameter (mPAD) derived from chest computer tomography (CT) is a useful predictor of pulmonary hypertension. We addressed the effect of OSA on pulmonary involvement and enlarged mPAD in adults with scleroderma.

**Methods:**

All participants underwent pulmonary function testing, carbon monoxide diffusion capacity, chest CT, and overnight sleep recording with home sleep apnea testing. OSA diagnosis was based on an apnea-hypopnea index (AHI) ≥ 15/h. Oxygen desaturation index (ODI) was also recorded. Scleroderma involvement of the lungs was defined as the Warrick score ≥ 7 based on the CT findings. Enlarged mPAD was defined as an mPAD ≥ 29 mm in men and ≥ 27 mm in women.

**Results:**

After exclusions, 62 patients (58 women) were included. OSA was found among 20 (32%), 17/42 (38%) in the limited cutaneous type, and 3/20 (15%) in the diffuse cutaneous type (*p* = 0.08). Scleroderma involvement of the lungs was observed in 40 participants (65% in OSA vs 64% in no-OSA; n.s.). Enlarged mPAD was measured in 16 participants, 10 of 20 (50%) in the OSA group and 6 of 17 (14%) in the no-OSA group (*p* = 0.003). OSA was associated with enlarged mPAD (odds ratio 4.7, 95% confidence interval 1.1–20.9; *p* = 0.042) independent of age, body mass index, and pulmonary involvement. There was a linear relationship between mPAD and AHI (*r* = 0.37; *p* = 0.003) as well as ODI (*r* = 0.41; *p* < 0.001).

**Conclusions:**

In this cohort, OSA was associated with risk for pulmonary hypertension independent of pulmonary involvement. These findings suggest that assessing the effect of therapy for concomitant OSA in patients with scleroderma is warranted.

**Trial registration:**

NCT 02740569

## Introduction

Scleroderma is an autoimmune, inflammatory, progressive, and multi-systemic disease, which is characterized with fibrosis in the skin and the connective tissue of the viscera. Scleroderma can affect the lungs, kidneys, gastrointestinal system, and heart [1]. When the lungs are affected, scleroderma is often progressive with high risk for morbidity and mortality [2]. Pulmonary involvement has been demonstrated among 70–90% of patients suffering from scleroderma [3].

Obstructive sleep apnea (OSA) is characterized by repeated apnea and hypopnea episodes and disturbances in arterial oxygen saturation [4]. It has been suggested that OSA is associated with local or systemic inflammatory diseases [5]. Less is known regarding the association of OSA with scleroderma. In one study, addressing the occurrence of OSA in interstitial lung disease, scleroderma constituted a small subgroup of 18 patients among others [6]. To our best knowledge, occurrence of OSA solely in patients with scleroderma with or without pulmonary involvement has yet not been defined. It is also unclear to which extent a concomitant OSA is associated with pulmonary hypertension in scleroderma patients.

Pulmonary hypertension is defined as a mean pulmonary artery pressure (mPAP) over 20 mmHg based on right heart catheterization [7]. However, right heart catheterization is invasive and demands exposure to contrast and ionizing radiation, and does not provide morphologic information [8]. Echocardiography is commonly used to screen suspected pulmonary hypertension patients [9], but the diagnostic accuracy of echocardiography is known to be dependent on several factors, such as body habitus, evident tricuspid regurgitation, heart rate, and the experiences of operators, which limit its clinical function [10]. Enlarged main pulmonary artery diameter (mPAD) ≥ 29 mm in men, and ≥ 27 mm in women, respectively, derived from chest computer tomography (CT), is suggested to be a useful predictor of pulmonary hypertension [11].

In the current study, we aimed to address occurrence of OSA in patients with scleroderma, and its possible association with CT-determined pulmonary involvement and enlarged mPAD.

## Methods

### Study design and patients

This cross-sectional cohort study was conducted at the Marmara University Pendik Teaching and Education Hospital, Istanbul, between 01 April and 01 October 2016. Patients with a known scleroderma diagnosis attending to the Departments of Rheumatology and/or Pulmonary Medicine, without any concomitant psychiatric or neurologic disease, were invited to participate. The study protocol conforms to the ethical guidelines of the 1975 Declaration of Helsinki as reflected in a priori approval by the Ethics Committee of the Medical Faculty of the Marmara University, Istanbul (approval no.: 09.2016.208). All patients provided written informed consent. The trial was registered with the ClinicalTrials.gov (NCT 02740569).

### Definition of baseline clinical characteristics

Anthropometrics, smoking habits, and past medical history for the whole population were obtained from the medical records. Height, weight, and waist circumference were measured, and body mass index (BMI) was calculated according to the formula body weight divided by height squared, and obesity was defined as a BMI of at least 30 kg/m^2^ [12]. Patients were classified as having limited cutaneous systemic sclerosis or diffuse cutaneous systemic sclerosis according to the criteria of Leroy et al., based on the extension of skin involvement [13]. Anti-nuclear antibody (ANA) was detected by using immunoflourescent assay (IFA) with a screening titer of 1:160 and above. The presence of anti-topoisomerase (anti-Scl70) and anti-centromere (ACA) were measured by using immunoblotting method—extractable nuclear antigen (ENA) panel antibodies. Information about concomitant diseases at baseline, including hypertension and diabetes, was based on self-reported and/or physician-diagnosed conditions. Ongoing medications were also obtained.

### Pulmonary function testing

Pulmonary function including forced expiratory volume in 1 s (FEV_1_), and forced vital capacity (FVC), was measured with MIR Spirolab II spirometry (Medical International Research, Rome, Italy), diffusing capacity of lung for carbon-monoxide (DL_CO_) in a body-plethysmograph (CareFusion Type MasterScreen PFT; Hoechberg, Germany), and a 6-min walking test (SMWT) was performed—in a 30-m flat indoor corridor—and all results were evaluated according to the ATS guidelines [14–17].

### High-resolution computed tomography

All patients underwent a high-resolution computed tomography (HRCT) at the Department of Radiology using Siemens SOMATOM Definition Flash (Erlangen, Germany) scans. A new HRCT was performed, if the existing measurement was older than 6 months. The scans of the chest were obtained with patients in supine position and with lungs at end-inspiration. The area of the scans ranged from the lung apices to bases, with 0.6-mm collimation, 1- to 2-mm slice thickness at 0.5-mm increments. Five crucial abnormalities were distinguished and their severity was assessed with the Warrick score [18]: ground glass opacities (score 1), irregularities in the pleural margins (score 2), septal and subpleural lines (score 3), honeycomb lung (score 4), and subpleural cysts (score 5). An additional extent score was added to each severity score, i.e., abnormality involving 1–3 segments (extent score 1), 4–9 segments (extent score 2), and more than 9 segments (extent score 3), respectively. A total score range was 0–30. Pulmonary involvement was defined as a Warrick score of at least 7 on the HRCT [19]. Explicitly, the transverse axial diameter of the mPA and the ascending aorta at the level of the bifurcation of the right pulmonary artery were measured [11]. The ratio of mPAD to ascending aorta diameter (ratio PA) was also calculated. All findings were scored by the same experienced pulmonologist (S.K.) blinded to the patients’ clinical characteristics and results of the pulmonary function tests and sleep recordings.

### Epworth Sleepiness Scale

Subjective sleepiness was evaluated using the Turkish version of the Epworth Sleepiness Scale (ESS) questionnaire, which is the most widely used index in sleep clinics [20, 21]. The ESS consists of 8 questions for assessing the chance of dozing off under 8 situations in the past month. Each item is scored from 0 to 3 (0, would never doze; 1, slight chance of dozing; 2, moderate change of dozing; 3, high chance of dozing). The ESS score ranges from 0 to 24. Excessive daytime sleepiness (EDS) was defined as the ESS score of at least 11 [22].

### Home sleep apnea testing

The portable, limited sleep study was performed with a home sleep apnea testing (HSAT) device (NOX-T3; Nox Medical Inc., Reykjavik, Iceland) and consisted of a nasal pressure detector using a nasal cannula/pressure transducer system, thoracoabdominal movement detection through two respiratory inductance plethysmography belts, and a finger pulse oximeter detecting heart rate and oxyhemoglobin saturation (SpO_2_) as well as body position and movement detection. Moreover, snoring was recorded by a built-in microphone in the NOX-T3 device which provides the opportunity to play the audio along with the other recorded signals during manual scoring of the events. The patient’s sleep time was estimated on the basis of self-reporting as well as the pattern of body movement during the study. Patients with an estimated sleep time of less than 4 h were offered a new HSAT. Apnea was defined as an almost complete (≥ 90%) cessation of airflow, and hypopnea was defined as a reduction in nasal pressure amplitude of ≥ 30% and/or thoracoabdominal movement ≥ 30% for ≥ 10 s if there was a significant oxyhemoglobin desaturation (decrease by at least 3% from the immediately preceding baseline value) according to the latest recommendations of the American Academy of Sleep Medicine [23]. Moreover, total number of significant desaturations was scored, and the oxygen desaturation index (ODI) was calculated as the number of significant desaturations per hour of estimated sleep. Minimum SpO_2_ and time spent below 90% SpO_2_ (TS90%) values were also recorded. OSA was defined as an apnea-hypopnea index (AHI) ≥ 15 events/h of the total estimated sleep time, based on the latest International Classification of Sleep Disorders -3[24], when OSA-related symptoms are absent. All HSATs were scored by the same physician (Y.P.), blinded to patient demographics, clinical characteristics, and results of the pulmonary function tests and HRCTs.

### Statistical analysis

Descriptive statistics are given as means ± SD and categorical variables as numbers (percentages). Comparison of categorical variables was done using the chi-squared test, and when appropriate, the Fisher exact test. Differences in means between groups were analyzed by Student *t* test, and when there was a skewed distribution, the Mann-Whitney *U* test was used. Pearson correlation coefficients were calculated for analyzing the associations between continuous variables of the pulmonary function tests, Warrick scores, mPAD values, and sleep recordings. A logistic regression analysis was used to test the association between OSA and pulmonary involvement as well as enlarged mPAD. All variables that were significant in the bivariate analyses were subsequently included in the multivariate model, and odds ratios (ORs) with 95% confidence intervals (CI) were calculated from the regression coefficients. All statistical tests were two-sided, and *p* < 0.05 was considered statistically significant. Statistical analysis was performed using the Statistical Package for Social Sciences, version 25.0 for Windows® system (SPSS® Inc., Chicago, IL, USA).

## Results

**Fig. 1:**
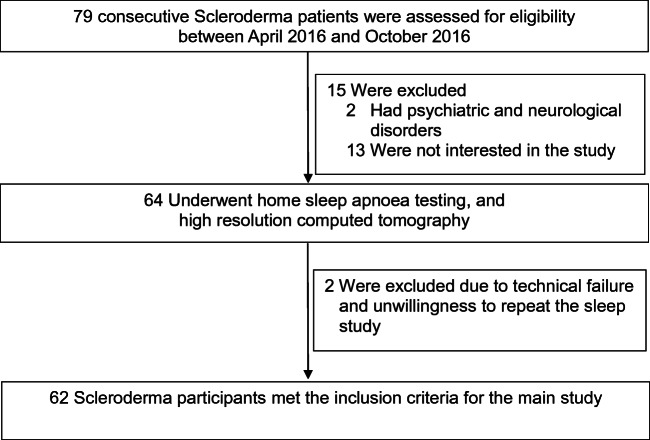
Flow chart of the study participants

As shown in Fig. 1, 79 patients with scleroderma were eligible for the study. Two patients were not included due to comorbid psychiatric and neurological diagnosis, and 13 patients refused participation. Out of 64 patients undergoing HSAT, 2 were excluded due to technical failure and their unwillingness to repeat the sleep studies. Thus, 62 patients (43 with limited scleroderma, 19 with diffuse scleroderma; mean age 48 ± 11 years (range 21–72 years)); 58 (93.5%) female; mean BMI 26.7 ± 5.0 kg/m^2^) with HSAT results and available HRCT scans within the last 6 months, were included in the final analysis.

As shown in Table 1, patients with the diffuse cutaneous scleroderma type were dominantly anti-Scl70-positive and had more pulmonary involvement based on the Warrick score compared with the limited cutaneous scleroderma type, but mPAD values did not differ significantly. AHI and ODI values were significantly higher in the limited sclerosis type.

**Table 1:** Characteristics of the study population based on the scleroderma type

	Limited cutaneous systemic sclerosis (*n* = 43)	Diffuse cutaneous systemic sclerosis (*n* = 19)	*p* value
Variables			
Age (years)	49.7 ± 12.3	45.8 ± 8.6	0.222
Age > 60, *n* (%)	10 (23.3)	1 (5.3)	0.149
Female, *n* (%)	42 (97.7)	16 (84.2)	0.082
BMI (kg/m^2^)	27.4 ± 4.6	25.2 ± 5.8	0.116
Obesity, *n* (%)	16 (37.2)	5 (26.3)	0.562
Current smoker, *n* (%)	3 (7.0)	1 (5.3)	1
Hypertension, *n* (%)	4 (9.5)	3 (15.8)	1
Diabetes mellitus, *n* (%)	3 (7.0)	2 (10.5)	0.638
Cardiac disease, *n* (%)	4 (9.3)	0 (0.0)	0.303
Disease duration (years)	7.2 ± 5.9	9.3 ± 7.0	0.219
AHI (events/h)	15.2 ± 13.4	7.6 ± 7.0	0.005
ODI (events/h)	13.1 ± 11.9	6.7 ± 6.0	0.007
Laboratory findings			
ANA titer ≥ 1/100, *n* (%)*	38 (90.5)	18 (100)	0.175
Anti-Scl70-positive, *n* (%)*	6 (14.3)	14 (77.8)	< 0.001
ACA-positive, *n* (%)*	10 (23.8)	1 (5.6)	0.148
HRCT, spirometry, DL_CO_ findings			
Warrick score	10.0 ± 9.4	17.1 ± 8.9	0.007
Warrick score ≥ 7, *n* (%)	24 (55.8)	16 (84.2)	0.031
mPAD (mm)	25.6 ± 5.5	25.2 ± 4.7	0.741
Enlarged mPAD, *n* (%)	13 (30.2)	3 (15.8)	0.347
DL_CO_ (%)**	72.6 ± 18.5	63.1 ± 22.7	0.097
DL_CO_ < 80%, *n* (%)**	27 (73.0)	16 (84.2)	0.507
Medications			
Corticosteroids, *n* (%)	13 (30.2)	9 (47.4)	0.194
Methotrexate, *n* (%)	5 (11.6)	4 (21.1)	0.941
Hydroxyklorokin, *n* (%)	27 (62.8)	12 (63.2)	0.978
Mycophenolate mofetil, *n* (%)	5 (11.6)	10 (52.6)	0.001
Azathiopyrin, *n* (%)	11 (25.6)	3 (15.8)	0.519
Leflunomide, *n* (%)	2 (4.7)	0 (0)	1

Values are mean ± standard deviation (compared using independent student *t* test) or number of patients (percentage) (compared using chi-squared test or Fisher’s exact test). *ACA*, anti-centromere; *AHI*, apnea-hypopnea index; *ANA*, anti-nuclear antibody; *Anti-Scl70*, anti-topoisomerase 1 antibody; *BMI*, body mass index; *DL*_*CO*_, diffusing capacity of lung for carbon-monoxide; *mPAD*, main pulmonary artery diameter; *ODI*, oxygen desaturation index. *Missing values in 2 patients. **Evaluated in 53 patients

In the entire cohort, OSA was observed among 20 (32.3%), 17 (38.1%) in the limited cutaneous type and 3 (15.0%) in the diffuse cutaneous type (Table 2). The participants with OSA were older and had higher BMI and waist circumference, and obesity and hypertension were more prevalent compared with those in the no-OSA group. Though the ESS score tended to be higher among the patients with OSA, the average value was quite low, and the predefined cutoff value of excessive daytime sleepiness (ESS score > 10) was observed among only 5 (25%) of the OSA patients (n.s.). The duration of the scleroderma since diagnosis as well as ongoing medications and inflammatory markers did not differ significantly between the groups at the time of the sleep studies. By definition, AHI and ODI values were higher, and SpO_2_ drops were more severe in the OSA group (Table 2). Proportion of the patients with pulmonary involvement based on the HRCT findings was similar in the OSA group (65.0%) compared with the patients without OSA (64.3%). There were no significant differences between the groups regarding FEV_1_%, FVC%, and DL_CO_% values and 6-min walking test. Enlarged mPAD was found among 16 participants, of whom 10 (50.0%) within the OSA group, and 6 (14.3%) in the no-OSA group (*p* = 0.003). The average mPAD as well as ascending aorta diameter were significantly higher in the OSA group while the ratio PA did not differ significantly (Table 2). The occurrence rate of enlarged mPAD was similar in the subgroup of patients with AHI < 15 but ≥ 5 (3 out of 23) compared with that among the patients with AHI < 5 (3 out of 19).

**Table 2:** Study population based on the results of the home sleep apnea testing

	OSA (−) (*n* = 42) (AHI < 15/h)	OSA (+) (*n* = 20) (AHI ≥ 15/h)	*p* value
Variables			
Age (years)	45.3 ± 11.4	55.14 ± 7.6	0.001
Age > 60, *n* (%)	5 (14.3)	6 (25.0)	0.302
Female, *n* (%)	39 (92.9)	19 (95.0)	0.748
BMI (kg/m^2^)	25.2 ± 4.6	29.9 ± 4.4	< 0.001
Obesity, *n* (%)	10 (23.8)	11 (55.0)	0.015
Waist circumference (cm)	92.3 ± 10.3	106 ± 9.5	< 0.001
Current smoker, *n* (%)	4 (9.5)	0 (0)	0.295
ESS score	4.9 ± 4.1	7.6 ± 5.7	0.041
Hypertension, *n* (%)	4 (9.5)	7 (35.0)	0.029
Diabetes mellitus, *n* (%)	3 (7.1)	2 (10.0)	0.654
Cardiac disease, *n* (%)	2 (4.8)	2 (10.0)	0.588
Disease duration (years)	7.2 ± 5.6	8.9 ± 7.3	0.334
Scleroderma type, diffuse cutaneous, *n* (%) limited cutaneous	17 (38.1) 26 (61.9)	3 (15.0) 17 (85.0)	0.082 0.082
HSAT findings			
AHI (events/h)	6.0 ± 4.3	27.2 ± 11	< 0.001
ODI (events/h)	5.3 ± 3.8	23.4 ± 10.4	< 0.001
Mean SpO_2_ (%)	94.9 ± 1.9	93.1 ± 1.7	< 0.001
Minimum SpO_2_ (%)	85.3 ± 6.8	81.7 ± 6.5	0.053
SpO_2_ < T90 (%)	3.3 ± 7.8	8.4 ± 14	0.070
Mean SpO_2_ drops (%)	3.4 ± 0.8	4.4 ± 0.8	< 0.001
HRCT, spirometry, and DL_CO_ findings			
Warrick score	12.1 ± 9.9	12.5 ± 9.8	0.882
Warrick score ≥ 7, *n* (%)	27 (64.3)	13 (65.0)	0.956
mPAD (mm)	24.2 ± 4.2	27.8 ± 5.3	0.006
Enlarged mPAD, *n* (%)	6 (14.3)	10 (50.0)	0.003
AAD (mm)	30.3 ± 4.9	34.0 ± 5.1	0.007
Ratio PA	0.8 ± 0.1	0.8 ± 0.2	0.590
DL_CO_ (%)*	67.2 ± 18.4	74.3 ± 26.0	0.267
DL_CO_ < %80, *n* (%)*	31 (81.6)	10 (66.7)	0.286
FVC/DL_CO_*	1.4 ± 0.4	1.2 ± 0.5	0.210
SMWT (m)	316.7 ± 74.5	296.9 ± 63.7	0.310

Values are mean± standard deviation (compared using independent student *t* test) or number of patients (percentage) (compared using chi-squared test). *AHI*, apnea-hypopnea index; *AAD*, ascending aorta diameter; *DL*_*CO*_, diffusing capacity of lung for carbon-monoxide; *ESS*, Epworth Sleepiness Scale; *FVC*, forced vital capacity; *HRCT*, high-resolution computed tomography; *HSAT*, home sleep apnea testing; *mPAD*, main pulmonary artery diameter; *ODI*, oxygen desaturation index; *OSA*, obstructive sleep apnea; *Ratio PA*, ratio of diameters mPA to AA; *SMWT*, 6-min walking test; *SpO*_*2*_, oxyhemoglobin saturation; *T90*, time spent below 90% oxyhemoglobin saturation. *Evaluated in 53 patients

**Fig. 2:**
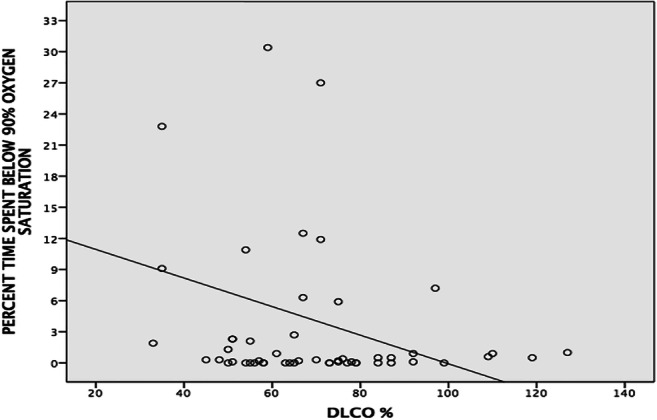
Correlation between percent time spent below 90% oxygen saturation and diffusing capacity of lung for carbon-monoxide (DL_CO_%)

In the logistic regression analysis, neither OSA nor the OSA indices (AHI, ODI, minimum SpO_2_, and TS90%) were associated with the CT-determined pulmonary involvement (data not shown). None of the OSA indices was significantly correlated with the Warrick score while there was an inverse relationship between TS90% and DL_CO_% (Fig. 2), and this relationship was independent of age, BMI, and AHI (β coefficient − 0.39, 95% confidence interval [CI] − 0.31–− 0.05; *p* = 0.007).

**Fig. 3:**
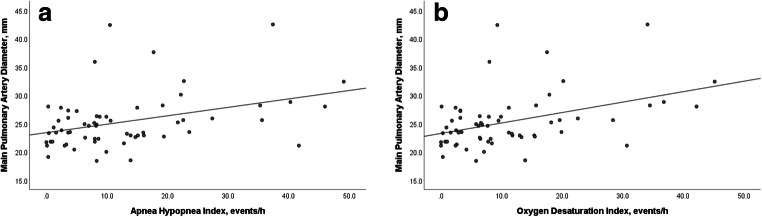
**a** Correlation between main pulmonary artery diameter and apnea-hypopnea-index. **b** Correlation between main pulmonary artery diameter and oxygen desaturation index

As shown in Table 3, BMI, OSA, and OSA severity indices were significantly associated with enlarged mPAD. In the multivariate model, OSA remained significant with a 4.7-fold risk for enlarged mPAD after adjustment for age, BMI, and CT-determined pulmonary involvement. Moreover, there was a significant linear correlation between mPAD and AHI as well as ODI (Fig. 3a, b).

**Table 3:** Variables associated with enlarged mPAD in adults with scleroderma in a logistic regression analysis

	Odds ratio	95% confidence interval	*p* value
Univariate			
Age	1.04	0.98–1.09	0.191
BMI	1.15	1.01–1.30	0.036
Obesity	2.54	0.79–8.19	0.119
Disease duration (years)	1.01	0.93–1.11	0.778
Hypertension	0.59	0.11–3.06	0.528
DL_CO_ (%)	0.97	0.93–1.00	0.083
DL_CO_ < 80%	2.13	0.41–11.06	0.369
Warrick score	1.01	0.96–1.07	0.671
Pulmonary involvement (Warrick score ≥ 7)	0.44	0.14–1.40	0.164
AHI ≥ 5/h	2.32	0.57–9.32	0.239
OSA (AHI ≥ 15/h)	6.00	1.75–20.55	0.004
AHI	1.07	1.02–1.13	0.006
ODI	1.09	1.03–1.16	0.004
Minimum SpO_2_ (%)	0.89	0.82–0.97	0.010
SpO_2_ < T90 (%)	1.05	1.00–1.11	0.065
Multivariate			
Age	1.01	0.94–1.07	0.878
BMI	1.07	0.93–1.24	0.360
Pulmonary involvement (Warrick score ≥ 7)	0.37	0.10–1.39	0.551
OSA (AHI ≥ 15/h)	4.70	1.06–20.88	0.042

*AHI*, apnea-hypopnea index; *BMI*, body mass index; *DL*_*CO*_, diffusing capacity of lung for carbon-monoxide; *ESS*, Epworth Sleepiness Scale; *mPAD*, main pulmonary artery diameter; *ODI*, oxygen desaturation index; *OSA*, obstructive sleep apnea; *SpO*_*2*_, oxyhemoglobin saturation; *T90*, time spent below 90% oxyhemoglobin saturation

## Discussion

In the current study, OSA was associated with risk for pulmonary hypertension in scleroderma patients independent of pulmonary involvement, and there was a significant association between mPAD and OSA severity indices, AHI and ODI.

To our best knowledge, this study is the first to report occurrence of OSA in a consecutive scleroderma cohort, mainly with the limited cutaneous scleroderma type, and its association with enlarged mPAD. One previous questionnaire-based study suggested that sleep disturbances were common in scleroderma patients, and this was mainly related with worsening of symptoms [25]. However, there were no OSA-specific questionnaires, and no sleep recordings were conducted in that report. The first study, in which the PSG parameters were presented, was conducted by Prado et al. who evaluated 27 scleroderma patients, and found no statistically significant difference between the assessed parameters including sleep stages but without any information about the respiratory events [26]. Other studies addressing the sleep disturbances in patients with scleroderma have suggested high occurrence of dyspnea, depression, serious reflux symptoms, pain, and itching during sleep, but without any information regarding OSA [25, 27].

In a previous study, addressing the relationship between OSA and interstitial lung disease, scleroderma constituted a subgroup of 18 adults among a total of 50 patients, and OSA was diagnosed among 10 of 18 participants with scleroderma (55.5%), applying an AHI cutoff value of 5 events/h according to the hypopnea criteria from 2007 [6]. Though not related with scleroderma, other studies have also addressed the relationship between parameters of OSA severity and interstitial lung disease with controversial results. In one study, PSG findings in idiopathic pulmonary fibrosis patients were evaluated, and the researchers found a significant correlation between AHI and DL_CO_ [28], whereas another study failed to demonstrate such an association [29]. In our cohort, we found a significant inverse linear relationship between DL_CO_ and TS90%, as also reported by the abovementioned study [6]. Interestingly, scleroderma patients with OSA had higher TS90% but also higher DL_CO_ values than those without OSA. This discrepancy may be explained by possible within-group differences in the OSA subgroup. Indeed, the vast majority of the OSA patients had limited cutaneous systemic sclerosis, and thus, less DL_CO_ reduction than those without OSA who had mainly the diffuse cutaneous form. The proportion of patients with DL_CO_ < 80% did not differ significantly between the groups, and the higher TS90% among the OSA patients may also indicate that these drops were mainly due to the OSA per se, independent of concomitant pulmonary involvement in patients with scleroderma.

To our best knowledge, there is yet no study addressing the association between OSA and pulmonary hypertension in patients with scleroderma. Previous reports from sleep clinic cohorts have suggested that nocturnal hypoxemia in moderate-to-severe OSA may contribute to vascular remodeling in pulmonary artery architecture, which may elevate pulmonary vascular resistance [30, 31]. In one previous study, evaluating 155 patients with resistant pulmonary hypertension patients who underwent sleep study, there was a significant association between OSA severity and mPAD [32].

Pulmonary hypertension is defined as a mPAP over 20 mmHg based on right heart catheterization [33]. However, right heart catheterization is invasive, demands exposure to contrast and ionizing radiation, and does not provide morphologic information [8]. Echocardiography is generally used to screen-suspected pulmonary hypertension patients [9], but the diagnostic accuracy of echocardiography has been reported to depend on several factors, which limit its clinical application [10]. Enlarged mPAD derived from chest CT, based on the sex-specific cutoff values used in the current study, is suggested to be a useful predictor of pulmonary hypertension, based on the results of the Framingham Heart Study with a healthy reference group (11). However, due to the limited data regarding the correlation of these cutoff values to the pathological conditions at that time (year 2012), the same authors emphasized that the evidence was limited for using these data as cutoff values [11]. In a meta-analysis, published 2 years later, including 2134 subjects, the sensitivity of the summary estimates for mPAD measurement in the diagnosis of pulmonary hypertension was 0.79 (95% CI 0.72–0.84), and specificity, 0.83 (95% CI 0.75–0.89), respectively, [34].

We applied a cutoff level of 7 for the Warrick score for a CT-based diagnosis of pulmonary involvement as previously suggested [19]. This level corresponded to the best compromise between sensitivity (0.60) and specificity (0.83), with a positive predictive value of 0.82 [19].

### Study limitations

First, the total number of patients in this single-center study is not sufficient for a proper prevalence study, and the results should be interpreted cautiously. However, scleroderma is a rare disease, and our clinical cohort is, indeed, to date, the largest sample in literature. Second, we did not perform a full-night PSG, and type 3–level HSAT might have resulted in an underestimation of OSA diagnosis due to overestimation of total sleep time. However, a cutoff level of AHI 15 events/h has been shown to be reliable for OSA diagnosis in the absence of OSA-related symptoms with this kind of diagnostic equipment [35]. Third, we did not perform right heart catheterization, which is the gold standard for the establishment of pulmonary hypertension diagnosis [33]. However, its risks and disadvantages have also been discussed [8]. Chest CT is routinely conducted for investigation of pulmonary involvement, and mPAD may be used in combination with echocardiography for further consideration of right heart catheterization in selected cases. Finally, a prospective follow-up study to evaluate the clinical significance of the used cutoff values for mPAD with regard to reproducibility as well as changes in response to CPAP treatment in the OSA subgroup could give better insights for the mechanisms involved in the development of pulmonary hypertension in patients with scleroderma.

## Conclusions

We conclude that OSA was associated with risk for pulmonary hypertension independent of pulmonary involvement in this cohort. Whether or not the treatment of concomitant OSA in patients with scleroderma reduces this risk needs to be further evaluated.
